# Mental Well-Being in UK Higher Education During Covid-19: Do Students Trust Universities and the Government?

**DOI:** 10.3389/fpubh.2021.646916

**Published:** 2021-04-26

**Authors:** Margaret Anne Defeyter, Paul B. Stretesky, Michael A. Long, Sinéad Furey, Christian Reynolds, Debbie Porteous, Alyson Dodd, Emily Mann, Anna Kemp, James Fox, Andrew McAnallen, Lara Gonçalves

**Affiliations:** ^1^Healthy Living Lab, Northumbria University, Newcastle upon Tyne, United Kingdom; ^2^Department of Sociology, Oklahoma State University, Stillwater, OK, United States; ^3^Ulster University Business School, Coleraine, United Kingdom; ^4^Centre for Food Policy, City University, London, United Kingdom; ^5^Department of Geography, Institute of Sustainable Food, University of Sheffield, Sheffield, United Kingdom; ^6^Student Union, Northumbria University, Newcastle upon Tyne, United Kingdom; ^7^Marketing Department, Northumbria University, Newcastle upon Tyne, United Kingdom; ^8^Student Union, Ulster University, Coleraine, United Kingdom

**Keywords:** food security, housing security, recreancy, ecological disaster, lockdown

## Abstract

This paper draws upon the concept of recreancy to examine the mental well-being of university students during the Covid-19 pandemic. Briefly, recreancy is loss of societal trust that results when institutional actors can no longer be counted on to perform their responsibilities. Our study of mental well-being and recreancy focuses on the role of universities and government regulators within the education sector. We surveyed 600 UK students attending 161 different public higher education providers in October 2020 during a time when many UK students were isolated in their residences and engaged in online learning. We assessed student well-being using the Short Warwick-Edinburgh Mental Well-being Scale (scored 7–35) and found the mean score to be 19.9 [95% confidence interval (CI) 19.6, 20.2]. This level of well-being indicates that a significant proportion of UK students face low levels of mental well-being. Structural equation modeling (SEM) analysis indicates that high recreancy—measured as a low trust in universities and the government—is associated with low levels of mental well-being across the student sample. While these findings are suggestive, they are also important and we suggest that government and university leaders should not only work to increase food and housing security during the Covid-19 pandemic, but also consider how to combat various sector trends that might intensify recreancy.

## Introduction

The negative impact of the Covid-19 pandemic on the mental well-being and mental health of university students is serious and a growing concern (1–3). Low levels of mental well-being can reduce motivation, diminish concentration and hinder academic attainment [(4); except see (5, 6)]. Moreover, low levels of student mental well-being can also be a major factor in self-harm and suicide ideation (7). Previous studies suggest that factors such as race, gender, age, and financial strain are likely associated with student mental well-being (8, 9). While there is strong reason to suspect that the impact of these established factors on well-being are intensified during the Covid-19 pandemic, few studies have examined university student mental well-being and the role of institutional trust during the Covid-19 pandemic. That is, the Covid-19 has served as a reminder that social institutions such as education cannot be counted on to attenuate what Brown [(10), p. 1] labels an “ecological disaster.” As a result, in this work we draw upon a social-psychological perspective to argue that contemporary studies of student mental well-being should account for student trust in their university and government to ensure their mental well-being during the Covid-19 pandemic. To make this connection we draw upon Freudenburg's [(11), p. 915–916] concept of recreancy that we employ by measuring perceptions of trust in universities and government regulators to understand risk management associated with low levels of student well-being during Covid-19. Specifically, recreancy is “a retrogression or failure to follow through on a duty or trust” [(11), p. 916]. Staying true to Freudenburg's original conception of recreancy we do not lay blame on any institutional actors. Instead, the purpose of this research is to determine whether and how student levels of trust in two important actors in the education sector during Covid-19 may impact student mental well-being.

The current research is divided into five sections. First, we examine the concept of recreancy to demonstrate how it is relevant to ecological disasters such as Covid-19. Next, we examine the literature on student well-being, situating the concept of recreancy alongside important predictors of well-being to propose a model of student well-being during Covid-19. Third, we explain data collection and methods for testing our model of student well-being. In that section we draw upon a survey of 600 students currently enrolled in universities across the UK. The fourth section of this manuscript describes the findings of the research. Specifically, we discover trust is correlated with mental well-being but also appears to be shaped by food and housing insecurity as well as social and economic circumstances. Finally, we conclude by suggesting that recreancy, as operationalized by asking whether students trust their university and the government, is likely to be a critical variable in studies of student well-being during ecological disasters such as the Covid-19 pandemic.

## Ecological Disasters and Recreancy

One view of the current pandemic is that it is an anthropogenically-driven ecological disaster that has arisen because of technological advances in agriculture. In short, the modern world provides an ideal environment for emerging pathogens that can lead to such disasters. Brown (10) explains:

As cities and farm operations grew, people and animals crowded closer together. The result was a new epidemiological order, in which zoonotic diseases—ones that could jump from animal to human—thrived. At first, these diseases remained confined to the places where they originated. [However]…infectious diseases have broken out more than twelve thousand times over the past three decades. It's no small feat to cross the species barrier; these numbers speak to the scale of our agricultural system.

Thus, the interconnectedness of biological lives makes it likely, if not inevitable, for pandemics such as Covid-19 to occur. In particular, those advances in agriculture technology have allowed for unprecedented levels of food production and, when combined, global travel and trade can contribute to the creation of an ecological network that binds us all together and lay the groundwork for ecological disasters (see (12, 13)).

It is within the context of ecological disaster that we draw upon Freudenburg's concept of recreancy [see also (14)]. Freudenburg (11) developed his theory of recreancy by drawing upon Durkheim's (15) theory of the division of labour, or the notion that societies are increasingly held together organically as occupational specialisation increases. While the division of labour is responsible for important technological advances, it is also simultaneously problematic (11). That is, “the very division of labour that permits many of the achievements of advanced industrial societies may also have the potential to become one of the most serious sources of risk and vulnerability” [(11), p. 914]. The implications of this unintended consequence specialisation are not only that technological disasters occur, but in Freudenburg's words that “natural forces” overcome institutional defenses that are no longer reliable. In short, social institutions are not trusted because institutional actors fail to carry out their obligations. While recreancy research tends to focus on the actors within institutions, Freudenburg believed in a more nuanced approach that linked these actors to their social institutions. Thus, Freudenburg (11, 16) conceived of recreancy as the deterioration or lack of trust in social institutions. This institutional focus allowed Freudenburg to maintain that recreancy was not about blaming institutional actors.

It is not relevant to know whether or not villainy can be discerned, whether at individual or collective levels; instead, to repeat Weber's words, the key question is simply whether experience shows that the behaviors of specialized individuals and institutions can be counted on [(11), p. 917].

We apply the concept of recreancy to the educational sector because it is often viewed as taking a major role in student “duty of care” and ensuring student well-being (17, 18). In short, the university has a direct impact on the lives of many students (19, 20). In the UK, universities have been under pressure for their response to Covid-19. For instance, the media has widely reported that students believe universities have failed to protect their well-being during lockdown (21–24). This pressure has led to a public outcry that the higher education sector cannot be trusted. For example, Manchester University was forced to publicly apologise “for the concern and distress caused” to students after university officials surrounded resident halls with guarded metal barriers during the night to keep students segregated (25). Anecdotally, students across the country have reported that they cannot count on universities during the Covid-19 crisis. As one student succinctly put it, “We were lied to” [(26), para 8]. Other students extend blame to government regulators who do not carry out their university oversight responsibilities and instead allow universities to freely take advantage of students. Moreover, some higher education advocates even suggest that the government has failed to provide universities with appropriate guidance and financing which leaves universities little choice but to exploit their own student populations. For example, one journalist observed, the “government has yet to show [universities] the sort of crisis support it tried to extend, for example, to the hospitality industry” [(26), para 7]. In the wake of these events students' advocate groups have called for additional help and students have engaged in organised protest activities ranging from rent strikes to virtual direct action by highlighting their grievances like food insecurity or prison-like living conditions to shame universities (22). More recently, students have organized a call for tuition and rent refunds as well as better access to campus facilities and student health and well-being support (22, 23, 27). In this research we suggest that whether the university and its regulators can be “counted on” during an ecological crisis such as Covid-19 has important implications for the mental well-being of students.

Unsurprisingly, there have been few studies of recreancy among university students. One notable exception is research by Ladd et al. [(28); see also (29)] into the relocation of nearly 50,000 New Orleans college students during Hurricane Katrina, a large Category 5 hurricane that struck southeastern United States in August 2005. Ladd et al. (28) discovered that students were filled with perceptions of recreancy, especially in relation to the government's response to the disaster. As the researchers report, “about six out of 10 students stated, based on their disaster experiences, they did not trust President Bush, FEMA (i.e., Federal Emergency Management Agency), the federal government, or the Louisiana state government” [(28), p. 64], with one university student summing up their feelings of recreancy as follows: “FEMA is a joke!” (p. 66). Students in the study reported that they “distrusted the federal government, even more than before” and could not “count on any politician.” While Ladd's study was appropriately focused on the trust of state and federal government response to relocating students during the Katrina disaster, we focus on recreancy by asking about trust in higher education and its operational response during Covid-19.

Despite the scarcity of research on student recreancy, the concept has been applied to a variety of technological and natural disasters (30–37). As Ritchie et al. [(36), p. 657] observe, recent scholars have noted, recreancy “offers important insights into social impacts such as loss of social capital and civility, as well as psychological responses of frustration, anger, and hostility frequently associated with these types of events” [see also (14, 38)]. While scholars have examined recreancy with respect to potential community impacts that disrupt and harm social relationship and create civil disorder there have been no studies, of which we are aware, that examine the concept of student recreancy during the Covid-19 pandemic. Thus, our examination of mental well-being is social-psychological in that we hypothesise that students experiencing high levels of recreancy, and therefore low levels of trust in the university and its regulators will also have lower levels of mental well-being than students who have high levels of trust in these two sets of actors.

## Predicting Student Mental Well-Being

The World Health Organization (39) states, “mental health is not just the absence of mental disorder [but] as a state of well-being in which every individual realizes his or her own potential, can cope with the normal stresses of life, can work.” Mental well-being is the experience of health and prosperity. It includes having good mental health, high life satisfaction, a sense of meaning or purpose, and an ability to manage stress (40).

In our review, we highlight research that directly measures well-being or its components, and mental health difficulties that could aid or disrupt an individual's potential. Previous research has overwhelmingly suggested that a variety of factors such as financial strain, gender, race and age, housing security and food security may impact well-being (9). We review these factors below prior to presenting our integrated model of student recreancy and well-being during Covid-19.

### Financial Strain

A number of studies have examined the economic circumstances and mental well-being of university students. Among the most studied variables are student financial pressures, which are likely to decrease mental well-being. For instance, university students who come from lower socioeconomic status households often face more financial strain and therefore have higher rates of mental health problems and lower levels of mental well-being than do those who come from more affluent households (41). In a study of Australian students, Stallman (42) found that students who identified as having any level of financial stress were much more likely to report decreased subjective mental well-being when compared to students with no financial stress [see also (43–45)]. In a recent UK study, Benson-Egglenton (46) found a clear relationship between students' mental well-being and financial circumstances. That is, students who faced financial hardship had lower levels of mental well-being. Benson-Egglenton reported that students who had higher well-being scores on the Short Warwick-Edinburgh Mental Well-being Scale (SWEMWBS) were less likely to need a student loan, more likely to receive financial support from their parents and less likely to be in debt when compared to those who had lower well-being scores.

### Gender

Male and female students have also been identified as having different levels of well-being. Female students are more likely to self-report symptoms consistent with mental illness than their male peers (41, 47, 48). In addition, female students are more likely than male students to perceive various academic, friend and work scenarios as stressful (49) which may impact mental well-being. Moreover, research on student well-being suggests that female students have lower levels of mental well-being than males and are also more likely to suffer from distress, including more somatic symptoms and anxiety/insomnia (47) which might be linked to academic performance. In particular, women in male-dominated fields of study are more likely to feel pressure to conforming to the gender stereotypes (i.e., “stereotype threat”), which is associated with poor mental health (50).

While considerable evidence exists that female students are more at risk of low levels of mental well-being than male students, a number of studies on gender and well-being are inconclusive. Lee and Loke (51) find that male students participate in more pro-health type behaviours than female students but that no gender differences in psychosocial well-being exist [(51); see also (52)]. Nevertheless, El Ansari et al. [(53), p. 293] found that even while females were more likely to rate well-being higher than males, they were also “more likely [than males] to feel psychosomatic/physical health problems … [and] … more likely to feel burdened overall.”

### Race/Ethnicity

White university students have higher levels of mental well-being (54) and lower levels of psychological distress (55) than other students. Wang and Castañeda-Sound (56) discovered ethnic minority students tended to feel less satisfied with life and experienced more stress than white students. Moreover, ethnic minority students often report having higher levels of stress and lower levels of mental well-being than white students, suggesting a potential correlation between stress and well-being (57, 58). The finding that ethnic minority students experience lower levels of mental well-being than white students is often reported in the literature, and there may be reasons for this finding other than stress (59–61). For instance, as is the case with stereotype threats faced by women, ethnic minority students may feel significant pressure to reject group stereotypes (62). Steele (63) discovered that being under threat of judgement by a racial stereotype leads to impaired performance on tests and is associated with lower levels of mental well-being. Other research suggests that ethnic minority students might experience low levels of mental well-being and higher levels of mental illness because of the university campus climate or existing institutional prejudice and discrimination (64–67). In a study of first year medical students Hardeman et al. (9) compared African American students to white students and found that African American students had nearly twice the risk of being classified as having symptoms of depression and anxiety. In short, the harmful social stereotypes and discrimination are likely to contribute to lower levels of mental well-being among non-white students.

### Age

Research suggests that young people are disproportionately impacted by low levels of mental well-being when compared to other ages (68). In addition, most studies of university student mental well-being that control for age suggest that students face a decline in their mental well-being in their first year of study (5). Older university students are more likely to seek help for mental health problems (41). While age seems to be a factor in mental well-being, some studies do not find a relationship between age and outcomes related to mental well-being, such as stress [e.g., (47)]. In addition, a few studies [e.g., (69, 70)] suggest there is a negative correlation among age and factors associated with mental well-being perhaps because older students (e.g., those typically in post-graduate school) are sometimes identified as being more sleep deprived (71) or are more likely to suffer from academic burnout (72). Finally, some research finds that age and gender may interact in that age only matters for female students, where older students report higher levels of mental well-being than younger students (73).

### Food/Housing Insecurity

Both food and housing insecurity are believed to be related (74) and predict low levels of mental well-being (75–82). Moreover, some students may even sacrifice basic food and housing needs to pay university tuition and fees. Food insecurity exists when there is insufficient or inappropriate access to food, while housing insecurity occurs when housing is unstable, unaffordable, unsafe or unavailable (83). There is growing recognition that food insecurity is tied to mental well-being on university campuses and many researchers are starting to conclude that food insecurity is likely to be a consistent and main factor associated with anxiety and depression among university students (84–87). A recent systematic review of 58 empirical studies from countries across the globe suggest that nearly one-third of university students may be food insecure and it is likely that that they suffer from “poorer nutritional outcomes, higher stress and depression and adverse learning, academic outcomes and/or productivity” as a consequence [(88), p. 1,780; see also (89)].

While housing insecurity is less studied than food insecurity among student populations it is, nevertheless, often mentioned in studies of student mental well-being (90). Moreover, in countries like the United States, 11–19% of undergraduate students are housing insecure [(91); see also (83)] and these rates are increasing (92). Importantly, Leung et al. (90) found that students who were facing housing insecurity were nearly twice as likely to report on a patient health questionnaire that they faced anxiety and depression, two conditions that negatively impact mental well-being.

Finally, it must be noted that food and housing insecurity are likely to impact well-being but are also likely to be strongly related to other important factors. For instance, financial strain is likely to have an important and direct impact on both housing and food insecurity (93–97) among students, which are also likely to impact mental well-being (98). Students who receive student loans are also more likely to be food insecure (74, 99) while those who have competing financial obligations are more likely to face food insecurity (100). Raskind et al. (98) found that students whose parents have less than a high school education, are receiving benefits and have lower discretionary budgets are more likely to identify as food insecure. Those studies that have been conducted suggest that poverty and financial stress leads to increased anxiety and poor mental health (41). Moreover, it is increasingly clear that marginalized students are particularly at risk. That is, non-white (101, 102), multiethnic (103), female [(95, 104), but see (98, 101)], Lesbian, Gay, Bisexual, Transgender, Queer (LGBTQ) students (105) are disproportionately food insecure when compared to white males.

## Methods

### Sampling and Data Collection

**Figure 1:**
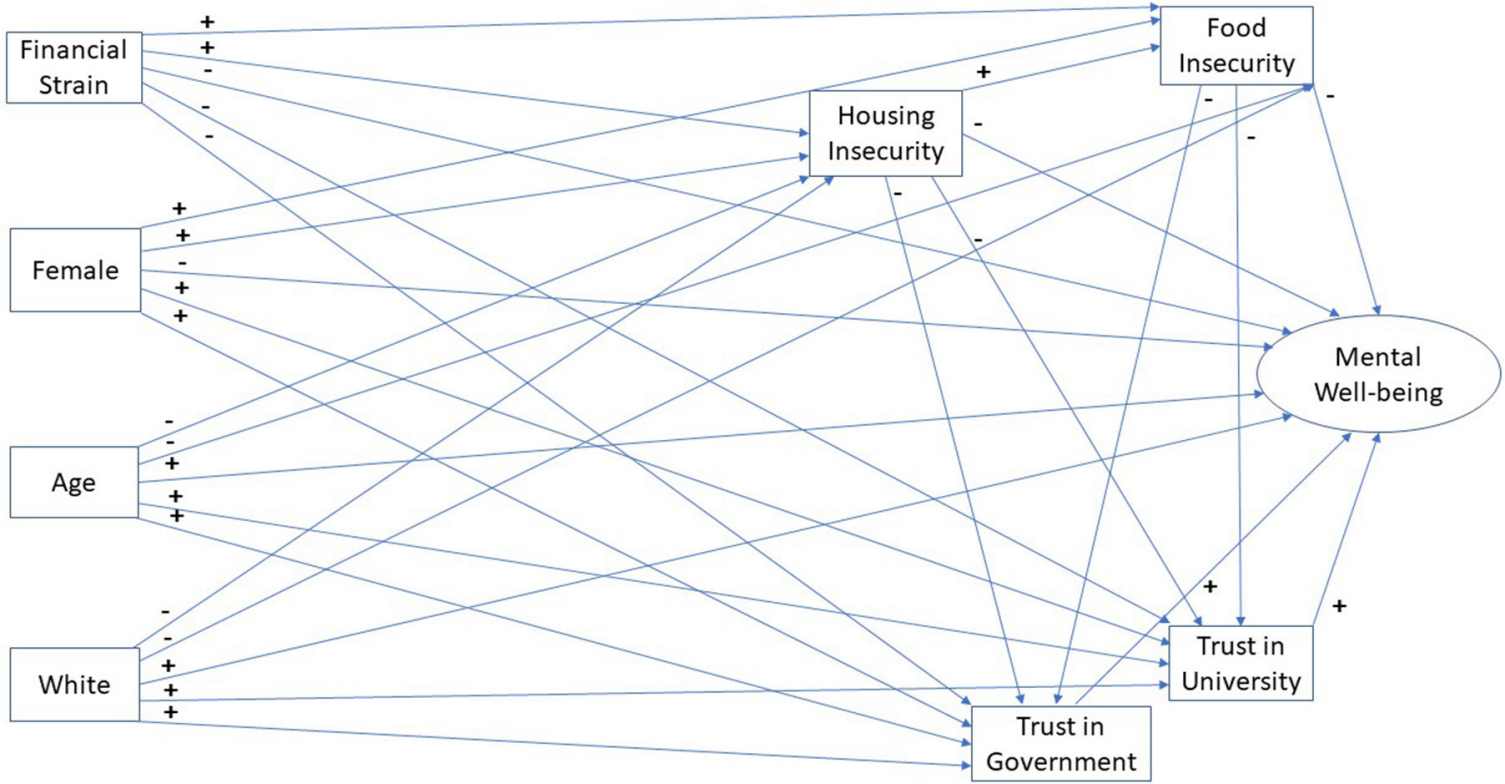
Conceptual model of university student mental well-being.

Research on recreancy and predictors of student mental well-being generated a set of hypotheses in Table 1 to be tested in this study. We are especially interested in examining the relationship between institutional trust and mental well-being within the context of the existing literature on student mental well-being. Figure 1 summarises the predicted relationships in the literature along with variables on institutional trust.

**Table 1:** Hypotheses (paths) tested in university student mental well-being model.

**Hypothesis**	**Selected Literature**
Financial Strain has a direct influence on mental well-being. Students who come from households that are financially strained are likely to face lower levels of mental well-being than students who come from households who have not faced economic disadvantage (H1).	El Ansari et al. (44), Benson-Egglenton (46), Eisenberg et al. (41), Lange and Byrd (43), Mulder and Cashin (45), and Stallman (42)
Gender has a direct influence on mental well-being. Female students will have lower levels of mental well-being than male students (H2).	Day and Livingstone (49), Eisenberg et al. (41), Saleh et al. (47), except see El Ansari and Stock (52), and Lee and Loke (51)
Race/Ethnicity has a direct influence on mental well-being. White students will have higher levels of mental well-being than other students (H3).	Aronson et al. (62), Ben-Ari and Gil (59), Blaine and Crocker (60), Cokley et al. (58), Dyrbye et al. (54), Griffith et al. (57), Hardeman et al. (9), Iwamasa and Kooreman (61), Prelow et al. (55), and Steele (63)
Age has a direct effect on mental well-being. Older students will have higher levels of mental well-being than younger students (H4).	Pedrelli et al. (68), except see Galbraith and Merrill (70), Saleh et al. (47), and Voltmer et al. (69)
Food and Housing Security will have a direct influence on mental well-being. Students who are food insecure will have lower levels of mental well-being (H5). Students who are housing insecure will have lower levels of mental well-being (H6).	Broton and Goldrick-Rab (78), Frongillo et al. (79), Heflin and Ziliak (75), Howell and Howell (76), Jones (80), Lee (81), Payne-Sturges et al. (74), and Stahre et al. (77)
Trust in Government will have a direct influence on student mental well-being. Students who trust the government to protect their health during the pandemic will have higher levels of well-being than students who do not trust the government to protect their health during Covid-19 (H7).	Freudenburg (11, 16)
Trust in their University will have a direct influence on student mental well-being. Students who trust their university to protect their health during the pandemic will have higher levels of mental well-being than students who do not trust their university to protect their health during Covid-19 (H8).	Freudenburg (11, 16)

The findings presented in this research are drawn from a cross-sectional sample of UK university students administered during the Covid-19 pandemic. Following ethical approval from the Faculty of Arts, Design and Social Sciences Ethics Committee at Northumbria University (reference number: 22790) a sample of 600 students was obtained with the help of *Prolific* (www.prolific.ac), an online survey platform that connects researchers to participants and is often used for social and economic research (106). Out of the 600 students who responded to the survey, 133 students did not provide answers to all the survey questions. As a result, the total sample size for this study is *n* = 467 students. We provide a breakdown of missing cases by variable in Appendix A (Supplementary Material) along with descriptive statistics for the variables included in our analysis (described below). Specifically, *Prolific* selected the student sample from a population of 4,758 eligible students who were immediately available to enroll in the research on a first-come, first-served basis. All participants received £1.50 compensation for their time to complete the short questionnaire that consisted of 38 close-ended questions. The questionnaire took <10 min to complete and was administered between 27 and 28 October 2020.

In 2018/2019 the UK Higher Education Statistics Agency reported that 2.38 million students were enrolled at 169 public higher education providers across England, Northern Ireland, Scotland and Wales. In the current study, the student sample consisted of 600 students from 161 public higher education and alternative providers in the UK. 93.5% of these students were undergraduates. Overall, the sample was 64% female (vs. 64% of undergraduates in the public university population in 2018/2019), 62% white (vs. 75% of undergraduates in the public university population in 2018/2019), 49% were under 21 years of age (vs. 57% in the undergraduate university population in 2018/2019), 22% report that they had received means-tested, free school meals during secondary education (vs. 19% who came from the most deprived areas of the UK in 2018/2019) and 45% reported that they were first generation HE students (vs. 50% in the university population in 2018/2019)^1^ Notable, then, the sample of students in this study appears to reflect the UK population of undergraduates with some amount of accuracy.

### Mental Well-Being

The primary dependent variable in the current study is mental well-being that is measured with the Short Warwick-Edinburgh Mental Well-being Scale (SWEMWBS). The SWEMWBS has been widely used by researchers studying mental well-being [e.g., (107–111)] and measures the positive aspects of mental health. The scale assesses mental well-being using a 5-point Likert scale (1 = “None of the time,” 2 = “Rarely,” 3 = “Some of the time,” 4 = “Often,” 5 = “All of the time”) on seven questions with an overall outcome score ranging from 7 to 35. All SWEMWBS scores were transformed using the published metric conversion recommended by Stewart-Brown et al. [(112), para 22]. Higher scores on the SWEMWBS are indicative of greater mental well-being. The SWEMWBS has been used to study student populations and is correlated with other scales measuring overall health, physical well-being, life satisfaction and emotional intelligence (108, 113, 114). Moreover, past research has found that in 2011 mean SWEMWBS scores for 16- to 24-year-olds in the English population ranged between 23.2 for women and 23.6 for men (108). The mean SWEMWBS score in the current sample is 19.9. While comparisons are difficult to make across diverse populations and time periods it is not surprising that the mean SWEMWBS score in the current sample is somewhat lower than reported in previous studies. Moreover, in the current study the SWEMWBS showed good internal consistency, with a Cronbach's alpha value of 0.86 in the sample. Appendix B in Supplementary Material lists the results of the confirmatory factor analysis for the mental well-being scale. As noted, the scale had factor loadings that ranged from 0.500 to 0.797.

### Recreancy

We measure recreancy as the amount of trust students place in their university and government to ensure their general well-being during the Covid-19 pandemic. To measure recreancy, we rely on two specific questions about trust: (1) “I trust the university to look after my well-being during the coronavirus pandemic” and (2) “I trust the UK government to ensure that my university will look after my well-being during the coronavirus pandemic.” Responses to these two questions are scored from strongly disagree = 1 to strongly agree = 5. In particular, the mean (median) for trust in the university is 3.35 (3.0) with 7.8% of students reporting that they strongly disagree that they trust that their university is working to ensure their well-being and 14.5% of students reporting that they strongly agree that they trust that their university is working to ensure their well-being. Overall, just over 25% of students disagree or strongly disagree that their university will look after their general well-being during the Covid-19 pandemic. The mean (median) scores for trust for government to regulate UK universities to promote student well-being is low as the mean score for this question is 2.3 (2). Nearly 31.7% of students strongly disagree that they trust the UK government to ensure their university will look after their general well-being while only 4.3% strongly agree that they trust the government to ensure that the university will look after their general well-being.

### Financial Strain

We use free school meal (FSM) status to identify students who are likely to come from households that are facing financial stain. In the England and Northern Ireland, pupils who are at least 7 years of age qualify for free school meals when the adults in the household claim one of several types of state benefits, including social security benefits in the form of income support, jobseeker's allowance, income related employment support, child tax credits, working tax credits and/or universal credit. In the case of universal credit, applicants must demonstrate an annual net earned income of £7,400 or less in England or £14,000 or less in Northern Ireland to receive FSM (115). While there are various potential measures of financial strain, Gorard [(116), p. 1,014] suggests that in the UK, using FSM as an indicator of poverty or financial hardship is “currently better than the alternatives…such as…household income, home resources, parental occupation(s) or social class.” Taylor (117) also suggests that while parental education, occupation and income are likely to be the best indicators of socio-economic disadvantage, researchers should be cautious about recommending replacing FSM eligibility for other alternative indicators of economic hardship as those indicators are often difficult to collect and the gain in predictive power is modest. In the present study we believe it is unlikely that many students would be unable to accurately report the household income of their parents and caregivers. As a result, we employ the relatively simple measure of FSM to identify those students who have come from households that are likely to face economic hardships. We measure financial strain by asking students whether they received FSM in their last year of secondary school. Students who come from households that face economic hardship are therefore eligible for FSM are also likely to face financial strains at university where they often rely on support from their family [see (46)]. Students scored “1” on the financial strain variable if they come from a household that received FSM in secondary school, while those who did not receive FSM were scored “0” on that variable.

### Gender

To capture the relationship between gender and mental well-being identified in the literature we measure gender using a dichotomous variable. Students were asked to report their gender (i.e., “female,” “male,” “non-binary,” “third gender,” or self-described). In our analysis female, non-binary, third gender, and self-described students were scored “1” while male students were scored “0.” As an alternative operationalisation of gender, we also compared female students (scored as “1”) to all other genders scored as “0.” We estimated a model for each operationalisation of gender and found that the models were nearly identical (not shown). That is, the alternative methods of measuring gender had no impact on this analysis as the coefficients, standard errors, and goodness of fit statistics were identical in both models.

### Race/Ethnicity

Students' Race/Ethnicity was measured using a 15-category nominal level variable. Results were largely clustered in White British category (i.e., White English/White Welsh/White Scottish/White Northern Irish/ White British) and spread evenly with relatively low frequencies (*n* = 4–23) among most other categories (e.g., African, Bangladeshi, Black British, Caribbean, Chinese, Indian, Pakistani, White, and Asian). As a result, we created the dichotomous variable where White UK students were scored 1 and students of all other races and ethnicities were scored 0. This variable therefore measures self-identified race/ethnicity categorized into white/non-white which likely is associated with social advantages.

### Age

Age is a ratio level variable that represents the student's age in years. The mean (median) student age was 23.0 (21.0) years old with a standard deviation of 6.5 years.

### Food Insecurity

Food insecurity was measured using the US Department of Agriculture's 6-item food security scale [see (95)]. The questions that made up the scale asked students to recall whether the following happened since the start of the Autumn 2020 term: (1) “The food that I bought just didn't last, and I didn't have money to get more”; (2) “I couldn't afford to eat balanced meals”; (3) “Did you cut the size of your meals or skip meals because there wasn't enough money for food?” and if “Yes”; (4) “how often did this happen?”; (5) “Did you ever eat less than you felt you should because there wasn't enough money for food?” and (6) “Were you hungry but didn't eat because there wasn't enough money for food?” The possible responses to questions 1 and 2 were “never,” “sometimes,” or “often,” while the responses to questions 3, 5, and 6 were “yes” or “no.” Finally, the responses to question 4 was “almost every month,” “some months but not every month,” or “only 1 or 2 months.” Responses of “often” or “sometimes” on questions 1 and 2, and “yes” on questions 3, 5, and 6 were scored as 1. Responses of “almost every month” and “some months but not every month” on question 5 were scored 1. All other non-missing answers were scored 0. The sum of these six items ranged from 0 (“food security” -−52.8% of all students) to 6 (“very low food security” -−7.1% of all students). The mean (median) food insecurity score was 1.4 (0). Cronbach's alpha for the food insecurity scale is 0.88, suggesting high internal consistency for this variable.

### Housing Insecurity

Housing insecurity was measured by asking students the extent to which they agreed with the following statement since the start of the Autumn 2020 school term: “I am finding it difficult to pay my rent or mortgage.” Responses to this item ranged from 1 = Strongly Disagree to 5 = Strongly Agree. The mean (median) housing insecurity score was 2.5 (2.0).

### Analytic Strategy

Building on previous research, the purpose of the current study is to present a conceptual model of student mental well-being during the Covid-19 pandemic. As previously suggested, we hypothesise that recreancy, measured as trust in the University and Central Government, play an important role in shaping levels of student mental well-being. To carry out our analysis we estimated the structural equation model (SEM) presented in Figure 1 testing the hypotheses described in Table 1. We choose to use SEM because the literature suggests the relationships between food security, housing security, gender, race, age and economic status are complex and can take various paths to mental well-being. In addition, we believe that the focus by UK students on food and housing security is central to predicting student trust in their university and the government. In short, the SEM provided us with a method to present relatively complex relationships where is more than one dependent variable in a parsimonious fashion.

The SEM was estimated using the Stata 15 sembuilder function for 467 students for whom all information was available. We use maximum likelihood estimations (without imputation or deletion). As previously noted, scales for food insecurity and mental well-being are acceptable. We assess the model fit using the Root Mean Square Error of Approximation (RMSEA) and the Comparative Fit Index (CFI).

## Results

The descriptive statistics and bivariate correlation coefficients for the variables and scales in the analysis are in Appendix A (Supplementary Material). Those bivariate correlations indicate that student mental well-being is correlated with the food insecurity scale and three variables (housing insecurity, trust in their university and trust in government). An increase in food insecurity or housing insecurity across the sample of students is associated with a decrease in mental well-being. In addition, as trust in their university or trust in the government to regulate their university increases across students, student mental well-being also increases. Despite previous research findings on race, gender, past financial strain and age, none of these variables are associated with mental well-being in those bivariate correlations. However, we do observe that female students are more likely to face housing insecurity than male students. We also find that white students are less likely to trust the government than non-white students. Finally, we observe that higher levels of food insecurity and housing insecurity are associated with lower levels of trust in the university and lower levels of trust in the government. In short, the bivariate correlations suggest that student trust in the university and government are important, if not critical, variables in predicting student mental well-being.

**Figure 2:**
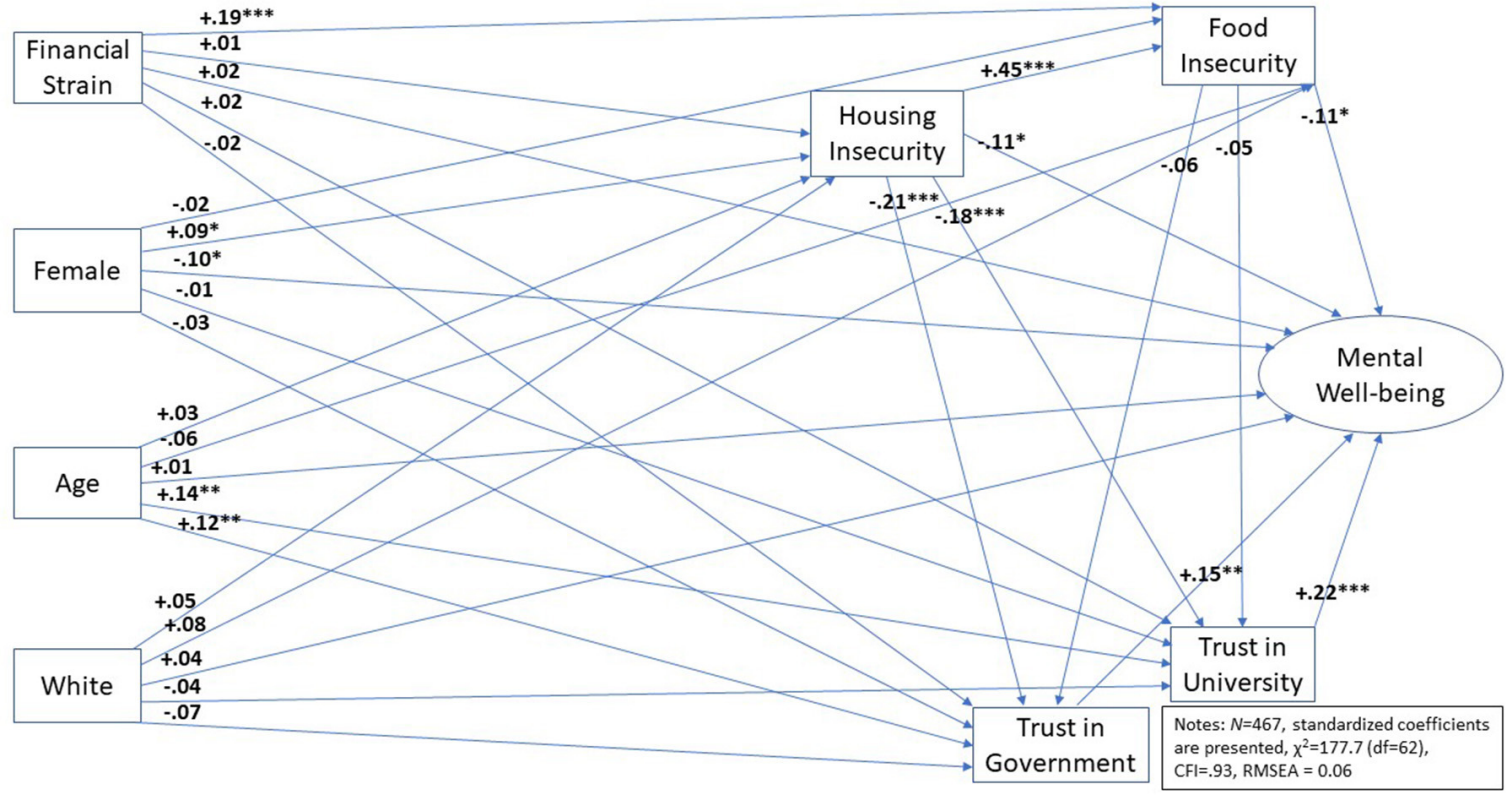
Empirical model of university student mental well-being. *p < 0.05, **p < 0.01, and ***p < 0.001.

Figure 2 presents the SEM hypothesised in Figure 1. Overall, the chi-square (χ^2^) for the model is 177.7, which is statistically significant (*p* < 0.05) and leads us to reject the null hypothesis that the observed and predicted models are equal. However, chi-square is highly sensitive to sample size and not recommended for use with samples as large as the one in the current study (118). As a result, we examine model goodness of fit using the comparative fit index (or CFI) and the root mean square error of approximation (or RMSEA). We choose the CFI because it is not sensitive to sample size and compares the fit of the observed model to the baseline model where all variables are uncorrelated (119). The CFI for the model in Figure 2 is 0.93, well above the acceptable benchmark value of 0.90 (120), equal to the value recommended by Byrne (121) and near the conservative benchmark of 0.95 recommended by Hu and Bentler (122). The RMSEA is a parsimony-adjusted absolute fit indicator that examines whether our specified model in Figure 2 reproduces the sample covariance matrix. The RMSEA for the model is 0.06, which is appropriately below the 0.08 benchmark value (122) and near the ideal 0.05 value recommended by Steiger (123). Finally, it is worth pointing out that when the chi-square statistic for model fit (χ^2^ = 177.7) is divided by the model degrees of freedom (*df* = 62) as a relative adjustment for sample size, the result is 2.87. This value is near the ideal value of 2 recommended by Ullman (124) well below the common cut-off value of 5 recommended by Schumacker and Lomax (120). In short, the model in Figure 2 appears reasonable.

The hypotheses presented in Table 1 are evaluated in Figure 2. When we examine the direct effects of financial strain, gender, age, and race/ethnicity on mental well-being (Hypotheses 1–4) we only find modest support for Hypothesis 2. That is, looking across students in the sample, female students tend to have slightly lower levels of mental well-being than male students (β = 0.10, *p* < 0.05). Turning to the relationship between food security, housing security and mental well-being (Hypotheses 5 and 6) we find that increasing levels of housing security are associated with decreased levels of mental well-being (β = −0.11, *p* < 0.05) and increasing levels of food insecurity are associated with decreasing levels of well-being (β = −0.11, *p* < 0.05). Thus, both hypotheses are supported.

Hypotheses 7 and 8 examine the impact of recreancy as measured through the variables trust in the university and trust in government university regulators. Figure 2 suggests that trust in the university is positively correlated with mental well-being. As students report that they trust their university to look after their mental well-being, their subjective well-being scores increase (β = 0.22, *p* < 0.05). The same relationship is found between government trust and mental well-being (β = 0.15, *p* < 0.05). Both relationships support hypotheses (H7 and H8) and suggest that trust has a negative association with student mental well-being. Moreover, student trust in their university and the government has two of the largest effects on mental well-being, suggesting that recreancy is an important aspect of student well-being during the Covid-19 pandemic.

## Discussion and Conclusion

There has been a recent call to investigate students' mental well-being during the Covid-19 pandemic (125). Although there have been several investigations into student well-being researchers have yet to examine the potential role of recreancy as measured by examining student perceptions of the failure of institutional actors such as universities and government regulators. As a result, there is a significant gap in current understandings of why some students may have particularly low levels of mental well-being during the Covid-19 pandemic. Our findings suggest that a lack of student trust in universities and government regulators may be an important factor in levels of mental well-being among students during ecological disasters. That is, recreancy appears to be important. While students have likely come to rely, at least partly, on university and government institutions to protect their mental well-being in the past, the perception by many students is that these actors can no longer be relied upon. Our analyses indicates that this form of recreancy could have an impact on student mental well-being.

Unfortunately, like most studies of student well-being our research suffers from some weaknesses. First, our sample is cross-sectional and does not consider how recreancy and mental well-being might have changed over time. As a result, it is difficult to say definitively whether levels of trust are impacted by Covid-19. We must point out, however, that there is pretty clear evidence that food insecurity and housing insecurity, things that should influence trust, have intensified during the Covid-19 pandemic [e.g., see (125–128)].

Second, the cross-sectional nature of our study means that it is not possible to establish causation. In particular, the association between mental well-being modeled in our data could be reversed, such that low levels of student mental well-being give way to low levels of trust. To examine this issue in more detail we tried alternative SEM models where mental well-being was used to predict trust (not shown). However, these efforts failed to produce a better fitting model. Thus, while our approach provides some empirical evidence that trust shapes mental well-being, more research is needed. That is, these findings need to be replicated in other settings and using longitudinal designs to better understand whether the relationship between trust mental well-being.

Third, as this is an observational study rather than experimental study it is possible that the association between mental well-being and trust could be confounded by an important third factor such as personality attributes or academic achievement. For instance, personality attributes such as neuroticism, extroversion, openness, agreeableness and conscientiousness may all influence levels of mental well-being and may also be related to how much faith and trust students place in the university and government during Covid-19. This study did not account for various personality factors that may influence mental well-being and as a result, as is the case with all observational studies, some caution must be exercised when interpreting results.

Fourth, our research is based in the UK, and the finding regarding demographic variables, food insecurity, and housing insecurity on mental well-being are largely consistent with the majority of studies on student mental health and mental well-being across the globe; it remains uncertain whether the mental well-being of higher education students in other countries would be similarly correlated with recreancy. In particular, the present survey was administered during a period of high infection rates and when UK students and young people were being blamed by politicians and media for spreading the virus (129, 130). The consequence of this “blame” may have created a unique situation where student trust or confidence was uniquely related to well-being. Moreover, trust in UK government was also at an all-time low in 2019 with 34% of the population stating that they “almost never” trust government (131). Thus, it is possible that these low levels of trust among the majority of the UK population is relatively unique, perhaps limiting the generalisability of the study results.

In the end, these results suggest that universities across the UK should pay more attention to the potential relationship between trust and mental well-being. Among the more consistent findings in the literature are our results concerning gender, previous financial strain, food security and housing security, all of which have been found to impact mental health and/or mental well-being. Our models also suggest that problems attributed to universities, failure to act such as food insecurity and housing insecurity may increase feelings of recreancy and reduce mental well-being. Thus, we encourage universities to pay particular attention to the relationship between trust, food insecurity, housing insecurity, gender, financial strain, and mental well-being. If these variables are related as we suggest then universities and government should ensure that students have sufficient and appropriate access to healthy, nutritious, and culturally appropriate food, especially during periods of lockdown or self-isolation when many students and their families may be struggling to source food. Moreover, governments and universities might also consider the role of housing insecurity in impacting trust and mental well-being. This is the case because many students report that they feel stuck paying for unaffordable contracts in residences in which they are confined (and unable to leave) and/or living in housing that is unsafe for vulnerable students given the overall numbers of students residing in a property. Finally, while additional investigations into student trust and mental well-being are needed, we suggest that universities and governments might, nevertheless, consider a communication strategy for improving trust among students to promote mental well-being, especially by noting how they are attenuating food and housing insecurity. Thus, even while we recognise the weaknesses associated with the current investigation, we also suggest that there is strong reason to want to promote gender equality, food, and housing security that are found to be associated with mental well-being among university students. If an outcome of these efforts is to increase student trust in institutional actors in the education sector, all the better.

## Data Availability Statement

The raw data supporting the conclusions of this article will be made available by the authors, without undue reservation.

## Ethics Statement

The studies involving human participants were reviewed and approved by the Ethics Committee in Arts, Design and Social Sciences at Northumbria University (Reference Number 22790). The participants provided their written informed consent to participate in this study.

## Author Contributions

MD contributed to the design, methods, and writing the manuscript. PS and ML contributed to the questionnaire design, data analysis, and writing the manuscript. SF, CR, DP, and AD contributed to the questionnaire design and editing the manuscript. AK, AM, and LG contributed to the questionnaire design and data collection. JF contributed to the data collection. EM contributed to the questionnaire design, data collection, and editing the manuscript. All authors contributed to the article and approved the submitted version.

## Conflict of Interest

The authors declare that the research was conducted in the absence of any commercial or financial relationships that could be construed as a potential conflict of interest.

## Footnotes

^1^Population estimates derived from *Higher Education Student Statistics: UK, 2018/19 – Student Numbers and Characteristics* published 20 January 2020. Available online at https://www.hesa.ac.uk/news/16-01-2020/sb255-higher-education-student-statistics/numbers.
