# Supplementary material for: Mental Well-Being in UK Higher Education During Covid-19: Do Students Trust Universities and the Government?
Source: Front Public Health. 2021 Apr 26;9:646916. doi: 10.3389/fpubh.2021.646916 (PMC8107392; doi:10.3389/fpubh.2021.646916)
Supplement: Supplementary file 1 [file Table_1.DOCX]

**Appendix A. Bivariate Correlations and Descriptive Statistics for Variables in the Study.**

|  | 1 | 2 | 3 | 4 | 5 | 6 | 7 | 8 | 9 |
| --- | --- | --- | --- | --- | --- | --- | --- | --- | --- |
| 1. Mental Well-Being | 1.00 |  |  |  |  |  |  |  |  |
| 2. Financial Strain | 0.00 | 1.00 |  |  |  |  |  |  |  |
| 3. White | 0.02 | -0.01 | 1.00 |  |  |  |  |  |  |
| 4. Female | -0.09 | 0.00 | 0.00 | 1.00 |  |  |  |  |  |
| 5. Age | 0.02 | 0.05 | 0.15* | 0.00 | 1.00 |  |  |  |  |
| 6. Food Insecurity | -0.15* | 0.17* | 0.08 | 0.02 | 0.05 | 1.00 |  |  |  |
| 7. Housing Insecurity | -0.19* | 0.02 | 0.04 | 0.10* | 0.03 | 0.45* | 1.00 |  |  |
| 8. Trust Their University | 0.28* | -0.03 | -0.07# | -0.02 | 0.09* | -0.17* | -0.20* | 1.00 |  |
| 9. Trust in Government | 0.24* | -0.07# | -0.08* | -0.03 | 0.07 | -0.17* | -0.20* | 0.51* | 1.00 |
|  |  |  |  |  |  |  |  |  |  |
| Mean | 19.93 | 0.22 | 0.62 | 0.65 | 22.95 | 2.35 | 2.43 | 3.35 | 2.27 |
| Median | 19.25 | 0.00 | 1.00 | 1.00 | 21.00 | 2.00 | 0.00 | 3.00 | 2.00 |
| Standard Deviation | 4.00 | 0.42 | 0.49 | 0.48 | 6.47 | 1.09 | 2.95 | 1.15 | 1.18 |
| Min. Score | 7.00 | 0.00 | 0.00 | 0.00 | 18.00 | 1.00 | 0.00 | 1.00 | 1.00 |
| Max Score | 35.00 | 1.00 | 1.00 | 1.00 | 68.00 | 4.00 | 8.48 | 5.00 | 5.00 |
| Missing Values | 2 | 2 | 2 | 4 | 6 | 68 | 44 | 7 | 10 |
|  |  |  |  |  |  |  |  |  |  |

* *p<*0.05; # *p<*0.10
