# Supplementary material for: Mental Well-Being in UK Higher Education During Covid-19: Do Students Trust Universities and the Government?
Source: Front Public Health. 2021 Apr 26;9:646916. doi: 10.3389/fpubh.2021.646916 (PMC8107392; doi:10.3389/fpubh.2021.646916)
Supplement: Supplementary file 2 [file Table_2.DOCX]

**Appendix B. Confirmatory Factor Analysis Results for the Measurement Model of Short Warwick-Edinburgh Mental Well-being Scale**

| Observed Variable – SWEMWBS | Mean | St. Deviation | Standardized Factor  Loading | α |
| --- | --- | --- | --- | --- |
| I’ve been feeling optimistic | 3.10 | 0.99 | 0.703 | 0.861 |
| I’ve been feeling useful | 3.02 | 0.98 | 0.709 |  |
| I’ve been feeling relaxed | 2.92 | 0.94 | 0.685 |  |
| I’ve been dealing with problems well | 3.25 | 0.97 | 0.744 |  |
| I’ve been thinking clearly | 3.30 | 0.96 | 0.797 |  |
| I’ve been feeling close to other people | 3.19 | 1.11 | 0.500 |  |
| I’ve been able to make up my own mind about things | 3.57 | 0.97 | 0.689 |  |
|  |  |  |  |  |
|  |  |  |  |  |
